# Meaning in Life in Palliative Cancer Care: Psychosocial and Existential Outcomes—A Systematic Review

**DOI:** 10.1177/08258597261426760

**Published:** 2026-02-28

**Authors:** Francisco Rodrigues-Fouto, Paulo Reis-Pina

**Affiliations:** 1 Center for Palliative Medicine, Faculty of Medicine, 37811 University of Lisbon, Lisboa, Portugal; 2 Acute Palliative Care Inpatient Unit, 679540 Local Health Unit of Santa Maria, Lisboa, Portugal

**Keywords:** attitude to death, emotional distress, existential psychology, neoplasms, palliative care, psychotherapy, quality of life, spirituality

## Abstract

**Background:**

Existential distress, marked by hopelessness, loss of meaning, and spiritual suffering, is prevalent among patients with advanced illness, and is associated with psychological burden and a wish to hasten death (WTHD).

**Purpose:**

This systematic review aimed to synthesize current evidence on meaning in life (MIL) in adult palliative care (PC) populations, focusing on its associations with quality of life (QOL), mental health, existential and spiritual well-being (SWB), and WTHD.

**Methods:**

MEDLINE, Web of Science, Scopus, and the Cochrane Library were searched for eligible studies (English, 2016-2024) involving adult cancer patients receiving PC. MIL was examined as a central intervention component or outcome. Risk of bias was assessed: findings were synthesized narratively. The review was registered in PROSPERO.

**Results:**

Eight studies (n = 1733 participants) were included: four cross-sectional, two randomized controlled trials, one longitudinal observational study, and one qualitative study. Several studies had small samples and substantial attrition. Risk of bias was high (n = 7), and moderate in one cross-sectional study. MIL was inversely associated with depression, anxiety, demoralization, and WTHD; and positively associated with QOL and SWB. MIL may also mediate psychological outcomes (eg, purpose, coherence, and personal values). However, heterogeneity in MIL conceptualization and measurement, combined with low methodological quality, limited comparability and certainty of findings.

**Conclusion:**

MIL may be relevant to psychosocial/existential outcomes in PC. Conclusions are constrained by a small and methodologically weak evidence base. Further high-quality, longitudinal research is needed before MIL-centered interventions can be recommended for routine clinical practice.

## Introduction

### Rationale

Advances in medicine and public health have extended life expectancy, increasing the prevalence of chronic, progressive, life-limiting conditions such as advanced cancer, neurodegenerative disorders, and end-stage organ failure.^1^ These shifts challenge healthcare systems to move beyond curative models and embrace holistic, person-centered approaches. Meeting not only physical but also psychological, social, spiritual, and existential needs has become essential, particularly at the end-of-life (EOL).

Palliative care (PC) explicitly aims to relieve suffering and improve quality of life (QOL) for patients and families facing life-threatening illness.^2^ However, access to PC remains uneven, particularly in low- and middle-income countries, compounding disparities in care.^3^ Beyond physical symptoms, many patients experience existential distress—feelings of hopelessness, purposelessness, and loss of meaning—that are closely associated with depression, demoralization, and the wish to hasten death (WTHD).^4,5^

Emerging evidence identifies meaning in life (MIL)—defined as the sense that life is coherent, purposeful, and significant,^6^—as a key protective factor in serious illness. is increasingly recognized as fundamental to emotional and spiritual resilience.^7^ As Viktor Frankl famously posited, suffering becomes intolerable when perceived as meaningless.^8^ In palliative settings, helping patients find or preserve meaning is not only therapeutic—it is an ethical imperative.

Several psychotherapeutic models, including Meaning-Centered Psychotherapy and Dignity Therapy seek to restore a sense of meaning, dignity, and legacy near the EOL.^5,9^ A 2016 systematic review of twelve studies reported that MIL-focused interventions in cancer patients improved various outcomes, including purpose, QOL, spiritual well-being (SWB), optimism, hopelessness, depression, and WTHD.^10^ However, the evidence base has expanded substantially since then and remains heterogeneous and fragmented. MIL is variably conceptualized—as a psychological construct, a spiritual dimension, or an existential need—and the outcomes assessed range widely across studies.

Moreover, many existing reviews have focused narrowly on specific interventions, without synthesizing the broader evidence on how MIL relates to core patient outcomes in PC. There remains a pressing need to clarify these relationships using a structured, quality-appraised, and updated synthesis. Addressing this gap is essential to inform more integrated, evidence-based existential support within PC.

### Objectives

This systematic review aimed to synthesize and appraise the empirical evidence on MIL-centered interventions in adult patients with advanced cancer receiving PC. Specifically, we sought to examine how MIL-based interventions relate to outcomes such as QOL, mental health, WTHD, and existential or SWB. While not exhaustive of all MIL research, our focus on interventional and directly MIL-centered studies allows for a more targeted understanding of clinical utility, and sets the stage for future trials and implementation research.

## Methods

This systematic review followed the recommendations of the “Cochrane Handbook for Systematic Reviews of Interventions,” ^11^ and was reported in accordance with the “Preferred Reporting Items for Systematic Reviews and Meta-Analyses” guidelines.^12^

### Eligibility Criteria

Selected articles met the following criteria:

*Participants*: Adult patients with advanced cancer receiving PC. Any definition of “advanced cancer” and PC used by the original authors was accepted, including early PC, hospice care, and EOL care.

Exposure/Intervention: Studies examining MIL either as a central intervention component or as a primary psychological construct, including its role as an exposure, outcome, correlate, or mediator.

*Comparators*: Any comparator, if applicable. Usual care was accepted as a control condition.

Outcomes: QOL, mental health, WTHD, and existential or SWB.

*Study design*: Quantitative, qualitative, and mixed-methods primary studies were eligible, including interventional, observational, and qualitative designs. Reviews, editorials, academic theses, and commentaries were excluded.

Articles not accessible through our Faculty's subscriptions were excluded.

### Information Sources

MEDLINE (via PubMed), Web of Science, Cochrane Library and Scopus were searched for articles published between January 1, 2016, and December 31, 2024. The final search was conducted on January 2, 2025.

This date range was chosen to capture the most recent decade of research and to reflect the growing integration of existential care into PC practice, while recognizing that earlier foundational studies may have been excluded.

### Search Strategy

The search combined free-text terms with database-specific subject headings: (“palliative care” OR “terminal care” OR “hospice care” OR “end-of-life care”) AND (cancer* OR malign* OR neoplasm*) AND (“meaning in life” OR “purpose in life” OR “meaning of life” OR “purpose of life”). Only English-language publications were included, and grey literature or trial registries were not searched, which may have introduced language or publication bias. Full database search strategies are provided in Supplementary Table 1.

### Selection Process

Both authors independently screened titles and abstracts, followed by full-text assessment. Disagreements were resolved by discussion and consensus. No automation tools were used.

### Data Collection Process

Data extraction was performed independently by two reviewers. Study authors were not contacted for additional information.

### Data Items

Extracted data included study characteristics, design, objectives, participant characteristics, MIL conceptualization, intervention/exposure details, outcome measures, and main findings.

### Study Risk of Bias Assessment

Risk of bias was assessed using the revised Cochrane Risk of Bias tool (RoB 2) for randomized controlled trials (RCT),^13^ and ROBINS-I V2 for observational studies.^14^ Visual summaries were generated using the Risk-of-Bias VISualization (Robvis) tool.^15^ The Joanna Briggs Institute checklists were used for analytical cross-sectional and qualitative studies.^16,17^ For qualitative studies, overall quality was calculated as the proportion of “Yes” items; because no standard cut-offs exist, thresholds (low <0.5; moderate 0.5-0.7; high >0.7) were defined a priori for consistent interpretation.

### Effect Measures

The effect measures reported by the original authors were accepted and used for both the synthesis and the presentation of results.

### Synthesis Methods

A meta-analysis was not conducted due to the limited number of studies and the heterogeneity of interventions and outcomes, which precluded meaningful quantitative synthesis. Findings were synthesized narratively by outcome domain.

Results were synthesized by predefined outcomes: the impact of MIL-centered interventions on: 1) QOL; 2) mental health; 3) WTHD; and 4) existential and SWB.

Summary tables were used to aid comparison: Table 1 details study characteristics; Table 2, main results; Tables 3–5, risk of bias by study type; and Table 6, certainty and confidence of evidence.

**Table 1.** Characteristics of the Included Studies (n = 8).

Authors; Country; Year	Study Design	Objective	Participants		Main Outcomes	Scales and Tools Used	Key Observations
			Intervention Group	Control Group			
Rullán et al; Spain; 2017^18^	Exploratory qualitative study	To understand the experiences of healthcare staff in assessing sources of distress related to feelings of loss of dignity in patients at the end of life	n = 124 advanced cancer patients (sex and ages - N/A)	N/A	Patients’ dignity-related distress and Healthcare professionals’ perceptions of using the PDI	PDI, HADS, ESAS, FACIT–Sp	Three professionals (psychologist, physician and nurse) and a medical student administered the PDI
Testoni et al; Italy; 2017^19^	Cross-sectional study	To examine the role of perceived MIL and representation of death on psychological distress, anxiety, and depression	-n = 219 healthy people (females 79%), mean age 21 ± 2.14 years -n = 30 cancer patients (females 66.7%), mean age 58 ± 10.8 years. Main cancers: 30% breast, 23.3% lung, 13.4% gynecological and 13.4% gastrointestinal	N/A	Psychological distress, Depression and anxiety levels, MIL and Representation of death as either a “passage” or “annihilation”	PMP, TDRS, HADS, DT	The study had two phases: (1) with healthy participants and (2) with cancer patients
Guerrero-Torrelles et al; Spain; 2017^20^	Cross-sectional study	To analyze and to propose a theoretical model of functional relationships among WTHD, MIL, depression and performance status	n = 101 (males 61.4%); mean age 61.7 ± 11 years; advanced cancer, mainly: 21.8% lung, 13.9% colon, 10.9% gastric and 8.9% pancreas	N/A	WTHD, MIL, Depression and Performance status	SAHD, KPS, ECOG-PS, POS, HADS, Barthel Index	N/A
Gravier et al; USA, Brazil, Chile, India and Jordan; 2019^21^	Prospective longitudinal multicenter observational study	To examine the differences in MIL across 5 countries and to identify factors associated with MIL	n = 728 (females 50.4%); mean age 57 years (range 19-85); advanced cancer, mainly: 21.6% gastrointestinal, 18% breast, 15.8% respiratory and 10.6% genitourinary	N/A	MIL, Depression and anxiety levels, Spiritual pain, Financial distress and Physical symptoms (pain, fatigue, drowsiness, dyspnea, insomnia)	MILS, ESAS, KPS, CAGE questionnaire	This is a pre-planned secondary analysis
Fraguell-Hernando et al; Spain; 2020^22^	Randomized controlled trial	To compare IMCP-PC to counselling-based psycho- therapy in patients receiving home palliative care	n = 24 (females 58.3%); average age 67.75 years; advanced cancer, mainly: 20.83% colorectal, 20.83% lung, 12.5% gynecological	n = 27 (males 55.6%); average age 67 years; advanced cancer, mainly: 22.22% lung, 14.81% gynecological, 11.11% colorectal	Depression and anxiety levels, Emotional distress and Demoralization	HADS, DED, DS-II, U Mann-Whitney’ test	N/A
Liu et al; China; 2020^23^	Cross-sectional study	To evaluate the effects of MIL and individual characteristics on dignity in patients with advanced cancer	n = 167 (males 52.1%); mean age 60.3 ± 11.7 years; advanced cancer, mainly: 26.3% lung, 23.4% colon/rectum and 11.4% breast	N/A	Dignity-related distress, MIL and Performance status	PDI, MILS, KPS	N/A
Kissane et al; Australia; 2023^24^	Randomized controlled trial	To examine the efficacy of MaP Therapy in promoting posttraumatic growth and meaningful life attitudes (choices and goal seeking) in people living with advanced cancer	n = 55 (female 72.7%); mean age 60.4 ± 11.0 years; advanced cancer, mainly: 37.2% breast, 22.6% gastrointestinal, 7.6% gynecological, 7.5% lung, 5.7% melanoma, 3.8% brain and 1.8% genitourinary	n = 52 (female 78.7%); mean age 63.2 ± 11.8 years; advanced cancer, mainly: 43.25% breast, 20.4% gastrointestinal, 13.6% lung, 6.8% genitourinary, 4.5% gynecological, 2.3% melanoma and 2.3% brain	Posttraumatic growth, Life attitudes, Spiritual well-being, Depression and anxiety levels and Demoralization	PTGI, LAP-R, MQOL-R, FACIT-Sp, PHQ-9, DS-II, DADDS, GAD-7 and Australian Karnofsky Performance Scale	The intervention group received MaP therapy and regular oncology/palliative care and the control group received usual care
Bernard et al; Switzerland; 2017^25^	Cross-sectional study	To explore the relationship between spirituality, MIL, WTHD and psychological distress, and their influence on the QOL in palliative care patients	n = 206 (females 51.4%); mean age 67.5 years; advanced cancer, mainly: 28.6% digestive, 22.3% pulmonary and 13.1% urological	N/A	MIL, Spiritual well-being, Religiosity, Depression and anxiety levels, WTHD, and QOL	SMILE, FACIT-Sp, HADS, SAHD, SQOLS, IIR	N/A

Legend: CAGE (C – Cutting Down, A – Annoyance by Criticism, G – Guilty Feeling, E – Eye-openers); DADDS, Death and Dying Distress Scale; DED, Detection of Emotional Distress; DS-II, Demoralization Scale II; Short DT, Distress Thermometer; ECOG-PS, Eastern Cooperative Oncology Group Performance Status; ESAS, Edmonton Symptom Assessment Scale; FACIT-Sp, Functional Assessment of Chronic Illness Therapy-Spiritual Well-Being Scale; GAD-7, Generalized Anxiety Disorder-7; HADS, Hospital Anxiety and Depression Scale; IIR, Idler Index of Religiosity; IMCP-PC, Individual Meaning-Centered Psychotherapy-Palliative Care; KPS, Karnofsky Performance Status; LAP-R, Life Attitude Profile; MaP, Meaning and Purpose; MIL, Meaning in Life; MILQ, Meaning in Life Questionnaire; MILS, Meaning in Life Scale; MQOL-R, McGill Quality of Life Scale Revised; PDI, Patient Dignity Inventory; PHQ-9, Patient Health Questionnaire-9; PMP, Personal Meaning Proﬁle; POS, Palliative Care Outcome Scale; PTGI, Posttraumatic Growth Inventory; QOL, Quality of Life; SAHD, Schedule of Attitudes Toward Hastened Death; SMILE, Schedule for Meaning in Life Evaluation; SQOLS, Single-Item Quality of Life Scale; TDRS, Death Representation Scale; USA, United States of America; WTHD, Wish to hasten death.

**Table 2.** Main Results of the Included Studies (n = 8).

Study	Main Results	Significance of Results	Key Methodological Limitations
Rullán et al; Spain; 2017 ^18^	– The initial experiences with the PDI on the part of the professionals led them to systematically administer the questionnaire as part of an interview instead of having patients ﬁll it out themselves in written form. – What started out as an evaluation very often led to a profound conversation on the MIL, dignity, and other sensitive, key issues related to the process of the illness.	– The PDI has intrinsic therapeutic value and is useful in clinical practice, and it is also a way of examining issues related to dignity and the MIL within the context of advanced-stage illness. – There is a need for studies that examine patient experiences through a PDI-based interview.	Qualitative design; small sample; limited transferability beyond context.
Testoni et al; Italy; 2017 ^19^	– The PMP was inversely correlated with depression (r = −0.50, *P* < .001) and anxiety (r = −0.46, *P* < .001), which mediated its effect on distress (*Distress Thermometer*: β = −0.26, *P* < .001). – Higher MIL was associated with lower psychological distress (r = −0.41, *P* < .001), anxiety (r = −0.46, *P* < .001), and depression (r = −0.50, *P* < .001), both in healthy participants and cancer patients. – Religious faith was associated with a stronger sense of MIL and a perception of death as a passage rather than annihilation (TDRS correlation with PMP-Religion: r = −0.62, *P* < .05). – Religious participants had higher PMP scores (4.84 ± 0.71) compared to nonreligious participants (4.36 ± 0.74, t = 4.71, df = 217, *P* < .001). – Cancer patients exhibited higher depression scores (t = −4.95, df = 247, *P* < .001) and were more likely to perceive death as annihilation (TDRS: t = −30.54, df = 247, *P* < .001) than healthy participants.	– Participants who represent death as a passage and have a strong perception of the MIL tend to report lower levels of distress, anxiety, and depression.	Small sample; cross-sectional design; moderate risk of bias; limited generalizability.
Guerrero-Torrelles et al; Spain; 2017 ^20^	– The WTHD correlated significantly (*P* < .01) with MIL (*r* = 0.60), performance status (*r* = 0.548), and depression (*r* = 0.397). – The structural equation modeling analysis showed no significant direct effect between performance status and the WTHD, but there was a significant total effect because of the mediation of depression and MIL (accounting for 76.5% of the mediation effect).	– Interventions to enhance MIL could help to address and/or prevent the emergence of a WTHD in this population.	Cross-sectional design; self-reported measures; no longitudinal follow-up; causal inference limited.
Gravier et al; USA, Brazil, Chile, India and Jordan; 2019 ^21^	– The median MIL score was 25/32 (interquartile range 23-28). – There was no significant difference in the total MIL score among 5 countries (*P* = .11), though there were differences in domain sub-scores. – In the univariate analysis, patients with higher intensity of physical symptoms by ESAS score (pain, fatigue, drowsiness, dyspnea, insomnia), depression, anxiety, spiritual pain, and financial distress had significantly lower MIL. – In the univariate analysis, patients with higher levels of education, who were married, and who had higher optimism had significantly higher MIL. – In the multivariate analysis, higher total MIL scores were significantly associated with greater optimism (*P* < .001), lower depression (*P* < .001), lower spiritual pain (*P* < .001), and lower financial distress (*P* < .001).	– Country of origin was not a determinant of MIL. – MIL was significantly associated with optimism, depression, spiritual pain, and financial distress, underscoring the multidimensional nature of this construct and potential opportunities for improvement in addressing MIL of patients with advanced cancer.	Observational design; serious risk of confounding; cultural variability not fully explored.
Fraguell-Hernando et al; Spain; 2020 ^22^	– Demoralization (despair) significantly decreased in the IMCP-PC group (*P* = .001, Cohen's d = 1.26, r = 0.51), while in the counseling group, the decrease was smaller (*P* = .021, Cohen's d = 0.46, r = 0.228). It was the only variable that significantly improved in the counselling group. – Anxiety (HADS-A) scores decreased significantly in the IMCP-PC group (*P* = .017, Cohen's d = 0.73, r = 0.34) but not in the counseling group (*P* = .094, d = 0.47, r = 0.23). – Depression (HADS-D) decreased significantly in the IMCP-PC group (*P* = .001, Cohen's d = 1.27, r = 0.53), whereas in the counseling group, no significant reduction was observed (*P* = .648, d = −0.06, r = −0.03). – Emotional distress decreased significantly in the IMCP-PC group (*P* = .024, Cohen's d = 0.63, r = 0.30) but not in the counseling group (*P* = .636, d = 0.15, r = 0.07). – No significant between-group differences in patient perception of treatment structure, focus, or length (*P* > .05). – However, IMCP-PC was rated significantly higher in helping patients find MIL (*P* < .01, U Mann-Whitney test).	– IMCP-PC was more effective than general counseling in reducing demoralization, anxiety, depression, and distress. – The effect was strongest for depression and anxiety, with moderate to large effect sizes. – IMCP-PC participants reported a stronger perceived impact on MIL, reinforcing the intervention's existential benefits.	Randomized controlled trial with small sample; high attrition; lack of blinding; high risk of bias.
Liu et al; China; 2020 ^23^	– Patients reported a mean of 4.2 ± 4.9 problems affecting their sense of dignity, the most common being “experiencing physically distressing symptoms (57.5%), followed by “feeling that I am a burden to others” (41.9%), and “not being able to carry out tasks associated with daily living” (37.7%). – A moderate negative correlation was found between the total PDI score and the total MILS score (r = −0.568, *P* < .01). – Significant correlations were found between the PDI score and the scores for the five MILS dimensions other than the acceptance of death (r = −0.029, *P* = .713). – The total dignity score had the strongest correlation with controlling one's life (r = −0.584, *P* < .01), followed by existential frustration (r = −0.440, *P* < .01), MIL and satisfaction in life (r = −0.361, *P* < .01), the will to seek meaning (r = −0.289, *P* < .01), and bearing suffering (r = −0.177, *P* = .022). – Weak to moderate negative correlations were also found between the MILS total score and the four PDI dimensions other than social support (r = −0.138, *P* = .075). – A strong negative correlation was observed between the total PDI score and the KPS score (r = −0.658, *P* < .01). – Dignity was negatively related to the KPS score, total MILS score, care venue, and age. – The results of the multiple linear regression analysis showed that 49.8% of dignity-related distress could be explained by MILS score, age, inpatient status, and KPS score.	– Self-perceived dignity is significantly negatively associated with MIL, age, inpatient status, and performance status. – The early recognition of risk factors for the loss of dignity and interventions to enhance MIL may prevent the loss of dignity in patients with advanced cancer.	Cross-sectional design; cultural context limits generalizability; correlational data only.
Kissane et al; Australia; 2023 ^24^	– PTGI scores improved significantly in the intervention group at post-treatment (t1: *P* = .006, d = 0.7, mean difference = 6.7, 95% CI [2.1, 11.3]) and remained higher at follow-up (t2: *P* = .073, d = 0.5, mean difference = 5.6, 95% CI [−0.6, 11.8]). – LAPR score increased significantly (t1: *P* = .007, d = 0.4, mean difference = 13.3, 95% CI [3.8, 22.9]). – Spiritual well-being (FACIT-Sp) score increased post-treatment (t1: *P* < .001, d = 0.4, mean difference = 6.1, 95% CI [3.2, 8.9]) and remained higher at follow-up (t2: *P* = .016, mean difference = 5.1, 95% CI [1.0, 9.2]). – Patients who completed all 6 sessions showed stronger improvements in PTGI (t1: *P* = .005, d = 0.7, mean difference = 7.7, 95% CI [2.5, 12.9]; t2: *P* = .11, d = 0.7, mean difference = 8.6, 95% CI [2.1, 15.0]), LAPR (t1: *P* < .001, d = 0.7, mean difference = 21.7, 95% CI [12.4, 31.0]; t2: *P* = .33, d = 0.3, mean difference = 15.3, 95% CI [1.3, 29.3]) and FACIT-Sp (t1: *P* < .001, d = 0.6, mean difference = 6.9, 95% CI [3.6, 10.2]; t2: *P* = .013, d = 0.2, mean difference = 5.8, 95% CI [1.2, 10.4]), compared to control arm at both t1 and t2. – Patients who completed all 6 sessions showed significant improvement, compared to the control arm at T1, on the QOL Existential symptoms (*P* = .008, d = 0.6, mean difference = 3.9, 95% CI [1.1, 6.7]) and on anxiety (*P* = .010, d = 0.7, mean difference = −2.9, 95% CI [−5.0, −0.7]). – For patients completing < 6 sessions, the only significant improvement was on FACIT at T1 (*P* = .004, d = 0.1, mean difference = 5.0, 95% CI [1.6, 8.3]).	– Meaning and Purpose Therapy was effective in improving posttraumatic growth, life attitudes and spiritual well-being in advanced cancer patients. – Improvements were strongest immediately post-treatment but weakened over time, suggesting the need for booster sessions. – Completing all 6 therapy sessions led to the most significant benefits, indicating that dose matters in psychotherapy for existential distress.	Randomized controlled trial with high attrition; lack of blinding; high risk of bias; limited statistical power.
Bernard et al; Switzerland; 2017 ^25^	– A significant negative relationship was found between spiritual well-being and psychological distress (*P* < .001) and the WTHD (*P* < .001). – MIL was negatively associated with psychological distress (*P* < .01) but not significantly associated with WTHD (*P* = .07). – Spiritual well-being and depression were the strongest predictors of perceived QOL, with higher spiritual well-being correlating with better QOL (β = 0.43, *P* < .001), while higher depression was linked to lower QOL (β = −0.52, *P* < .001).	– Both spiritual well-being and MIL appear to be potential protective factors against psychological distress at the end of life. – Since nonphysical determinants play a major role in shaping QOL at the end of life, there is a need for the development of meaning-oriented and spiritual care interventions tailored to the fragility of palliative patients.	Cross-sectional design; self-reported outcomes; residual confounding; no longitudinal follow-up.

Legend: CI, Confidence Interval; ESAS, Edmonton Symptom Assessment Scale; HADS, Hospital Anxiety Depression Scale; IMCP-PC, Individual Meaning-Centered Psychotherapy-Palliative Care; KPS, Karnofsky Performance Status; LAPR, Life attitude profile-revised; MIL, Meaning In Life; MILS, MIL Scale; PDI, Patient Dignity Inventory; PGDS-PF, Prolonged Grief Disorder Scale-Patient Form; PMP, Personal Meaning Proﬁle; PRGI, Posttraumatic growth; QOL, Quality of Life; TDRS, Death Representation Scale; USA, United States of America; WTHD, Wish to hasten death.

**Table 3.**
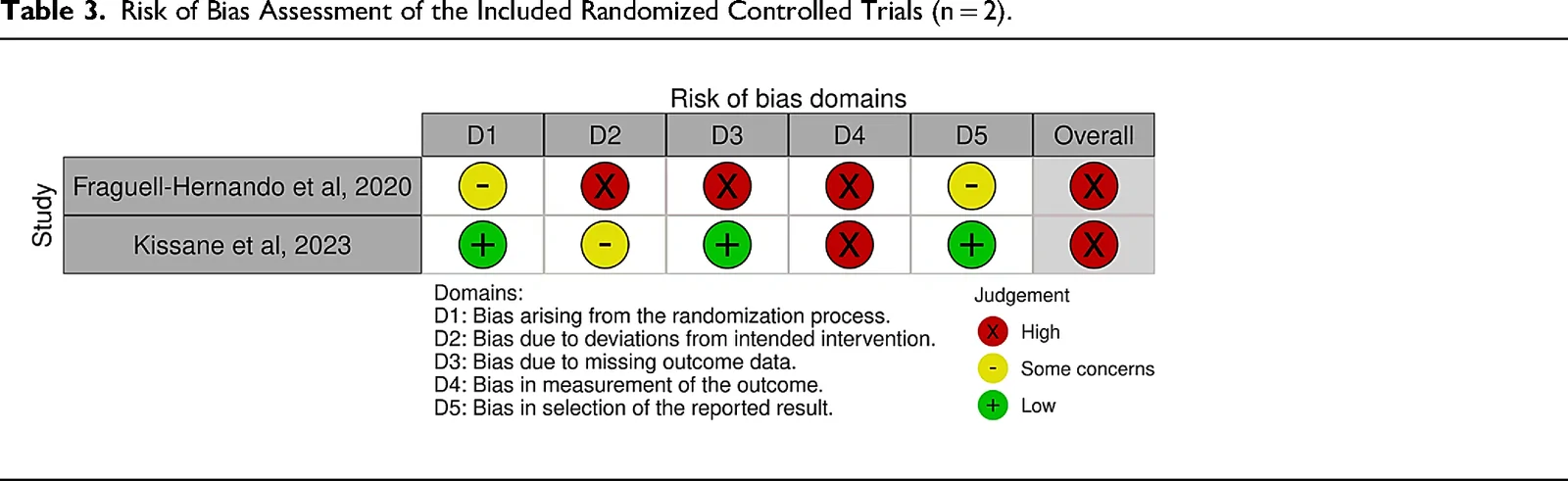
Risk of Bias Assessment of the Included Randomized Controlled Trials (n = 2).

**Table 4.**
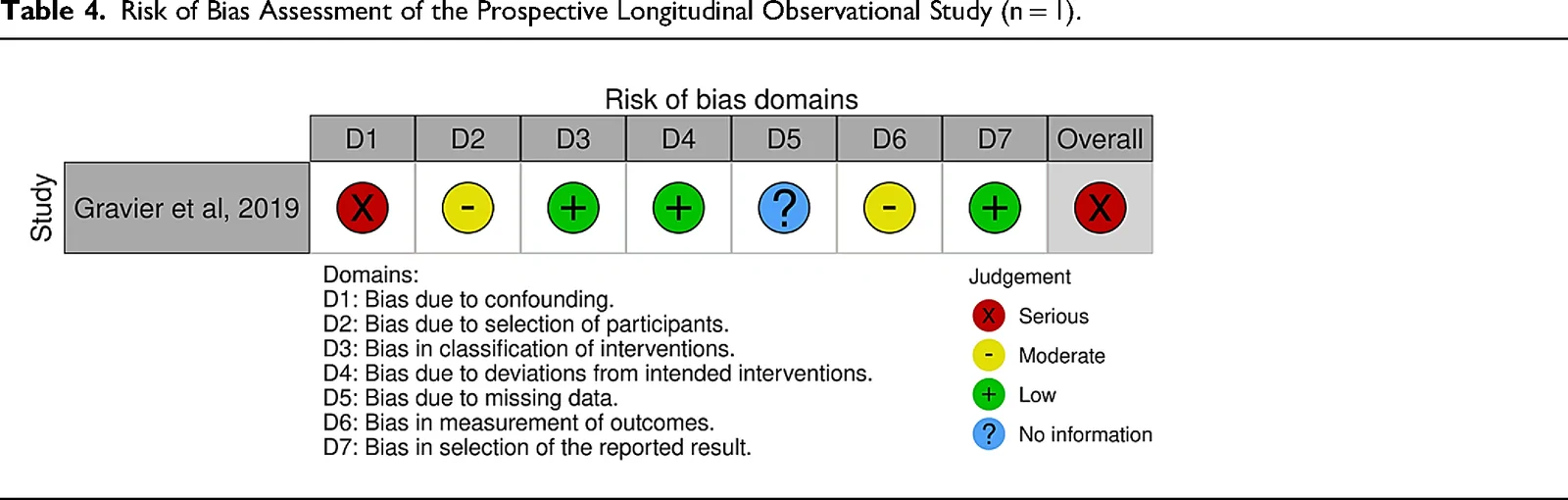
Risk of Bias Assessment of the Prospective Longitudinal Observational Study (n = 1).

**Table 5.** Risk of Bias of Qualitative and Cross-Sectional Studies (n = 5).

Authors; Country; Year	Total Number of Items	Number of “*Yes*”	Number of “*No*”	Number of “*Unclear*”	Number of “*Not Applicable*”	Degree of Quality	Overall Appraisal
Rullán et al.; Spain; 2017 ^18^	10	8	0	2	0	High	Include
Testoni et al.; Italy; 2017 ^19^	8	5	2	1	0	Moderate	Include
Guerrero-Torrelles et al.; Spain; 2017 ^20^	8	8	0	0	0	High	Include
Liu et al.; China; 2020 ^23^	8	8	0	0	0	High	Include
Bernard et al.; Switzerland; 2017 ^25^	8	6	0	2	0	High	Include

**Table 6.** Certainty and Confidence of Evidence: Summary of Findings (n = 8).

Outcome/ Review Finding	Contributing Evidence (References)	Study Designs	Risk of Bias/ Limitations	Inconsistency/ Coherence	Indirectness/ Relevance	Imprecision/ Adequacy	Publication Bias	Confidence/ Certainty
Spiritual Well-Being	22-25,27,28	2 RCT, 4 Observational	High–Moderate	No serious	No serious	Serious	Likely	Low (GRADE)
Mental Health (Anxiety, Depression, Distress)	22-25,27	2 RCT, 3 Observational	High (RCT), Moderate–Serious	Some	No serious	Serious	Likely	Very Low (GRADE)
Wish to Hasten Death	22,23,26,28	3 Cross-sectional, 1 Observational	Moderate	No serious	Some	Serious	Likely	Low (GRADE)
Quality of Life	25,27,28	2 RCT, 1 Cross-sectional	High (RCT), Moderate	No serious	No serious	Serious	Possible	Low (GRADE)
The PDI interview assists Meaning in Life dialogues.	21	Qualitative	Minor	High	High	Moderate	Not applicable	Moderate confidence (GRADE-CERQual)

Legend: GRADE, Grading of Recommendations Assessment, Development and Evaluation; GRADE-CERQual, Confidence in the Evidence from Reviews of Qualitative research; PDI, Patient Dignity Inventory; RCT, Randomized Controlled Trials.

### Certainty Assessment

Certainty and confidence of evidence were assessed separately for quantitative and qualitative findings. Two reviewers independently applied the “Grading of Recommendations Assessment, Development and Evaluation” (GRADE) approach to evaluate the certainty of evidence for quantitative outcomes.^26^ Certainty ratings began at “high” for RCT and “low” for observational studies, and were rated down based on risk of bias, inconsistency, indirectness, imprecision, and publication bias. For the qualitative study, we used the “Confidence in the Evidence from Reviews of Qualitative research” (GRADE-CERQual) approach,^27,28^ assessing confidence based on methodological limitations, coherence, adequacy of data, and relevance. Narrative summary-of-findings statements were produced for all domains, as most studies did not report variances or provide effect estimates compatible with meta-analysis (Table 6).

GRADE and GRADE-CERQual assessments were applied post hoc. This methodological decision was made to enhance interpretability but is acknowledged as a limitation of the review.

## Results

### Study Selection

**Figure 1.**
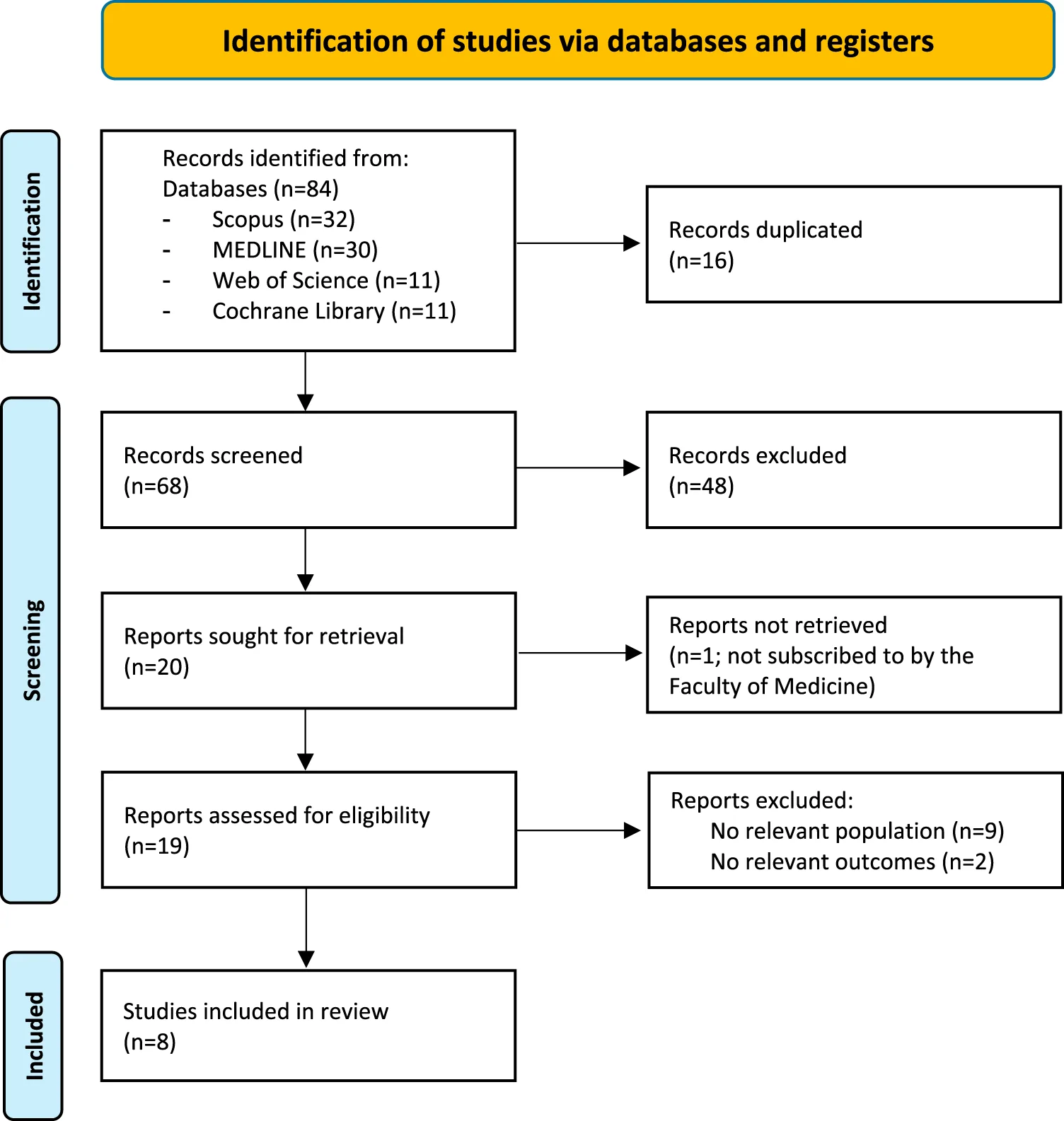
Flow diagram of the study selection process.

Eighty-four studies were identified. After removing 16 duplicates, 68 records were screened. Forty-eight were excluded based on title and abstract screening, leaving 20 for full-text review. One could not be retrieved due to subscription restrictions. Of the 19 assessed, 11 were excluded: nine for involving irrelevant populations (eg, healthcare professionals, students, or patients without advanced cancer), and two for evaluating non-aligned outcomes. Eight studies met all inclusion criteria and were included.^18–25^ The study selection process is summarized in Figure 1.

### Study Characteristics

Eight studies were included: three from Spain, and one each from Italy, Switzerland, China, Australia, and a multicenter study across the United States of America, Brazil, Chile, India, and Jordan. Study designs comprised four cross-sectional studies,^19,20,23,25^ two RCT,^22,24^ one prospective longitudinal observational study,^21^ and one qualitative study.^18^ Across all studies, there were 1733 participants: 1514 advanced cancer patients and 219 healthy individuals. Table 1 summarizes the characteristics of the included studies.

### Risk of Bias

All studies were assessed as having a high risk of bias, except Testoni et al.,^19^ which was rated as having a moderate risk of bias.

#### Randomized Controlled Trials

Fraguell-Hernando et al. were rated as high risk of bias (Table 3).^22^ Major concerns included insufficient details on randomization and allocation concealment, significant deviations from intended interventions (per-protocol analysis with notable non-adherence), high attrition (37% did not complete the study), and subjective self-reported outcomes without blinding.

Kissane et al. were also judged as high risk.^24^ While the randomization process and handling of missing data (via multiple imputation) were sound, the lack of blinding among participants and therapists raised concerns about deviations from the intended intervention and subjective outcome reporting, which likely influenced responses.

#### Observational Study

Gravier et al. were assessed as having a serious overall risk of bias, primarily due to confounding (Table 4).^21^ Key determinants of MIL (eg, performance status, social support, QOL) were not included in the analysis. Moderate risk was noted in participant selection and outcome measurement. Information on missing data was insufficient.

#### Cross-Sectional Studies

Guerrero-Torrelles et al. were rated high quality (Table 5).^20^ The study had well-defined inclusion criteria, valid tools, and appropriate statistical methods, including structural equation modeling. Limitations included its cross-sectional design and reliance on a single-item MIL measure.

Testoni et al. were rated moderate quality.^19^ Inclusion criteria for cancer patients were clear, but less so for healthy participants. While settings, measures, and tools were generally appropriate, confounding factors were not fully addressed.

Liu et al. achieved high quality, meeting all eight criteria.^23^ The study clearly described its population, used valid measures, identified confounders, and applied sound statistical techniques.

Bernard et al. were rated high quality, though identification and handling of confounders were rated “unclear.”^25^ Inclusion criteria, measurements, and statistical analyses (including regression and Bonferroni correction) were appropriate.

#### Qualitative Study

Rullán et al. were also rated high quality (Table 5).^18^ There was strong alignment between philosophical framework, methodology, and analysis. Participants’ voices were well represented through direct quotes, and ethical approval was obtained. Areas for improvement included a more explicit statement on the researchers’ theoretical positioning and influence on the research process.

### Results of Individual Studies

The results of individual studies are summarized in Table 2.

### Results of Syntheses

#### “Meaning in Life”-Centered Interventions and Quality of Life

Three of the eight included studies examined QOL in relation to MIL-centered interventions.^22,24,25^ Across these studies, SWB was consistently associated with higher QOL. Bernard et al. reported that SWB was a strong positive predictor of QOL (β = 0.43, *P* < .001), whereas depression was inversely associated with QOL (β = −0.52, *P* < .001).^25^ However, the cross-sectional design precludes causal inference.

Kissane et al. observed improvements in posttraumatic growth, life attitude, and SWB following a MIL-centered intervention, indicating changes in psychological and existential outcomes over time.^24^ Similarly, Fraguell-Hernando et al. found that participants receiving “Individual Meaning-Centered Psychotherapy-Palliative Care” (IMCP-PC) reported significant improvements in perceived MIL (*P* < .01) and emotional outcomes.^22^ These findings suggest a possible relationship between MIL-focused interventions and QOL-related domains rather than definitive treatment effects. Interpretation of these results is limited by important methodological constraints. Bernard et al. relied on correlational data,^25^ while both RCT were rated as high risk of bias due to substantial attrition, lack of blinding, and small sample sizes.^22,24^

Applying the GRADE framework, the certainty of evidence for QOL was rated as very low, driven by serious concerns regarding risk of bias, indirectness, and imprecision (Table 6). Accordingly, although the findings are consistent with a potential association between MIL-centered approaches and improved QOL-related outcomes, conclusions must remain cautious and tentative.

#### “Meaning in Life”-Centered Interventions and Mental Health

Six of the eight included studies examined the relationship between MIL and mental health outcomes.^19–22^^,24,25^ In cross-sectional analyses, Testoni et al.^19^ reported strong inverse correlations between MIL and both depression and anxiety (r = −0.50, *P* < .001), while Bernard et al.^25^ found that higher SWB was associated with lower psychological distress (*P* < .001). Similarly, in a large multinational study, Gravier et al. found that higher levels of depression (estimate = −0.45, *P* < .001) and anxiety (estimate = −0.36, *P* < .001) were significantly associated with lower MIL in univariate analyses.^21^ These findings indicate consistent associations but do not permit conclusions about directionality or causation. Guerrero-Torrelles et al. further reported that depression was positively correlated with WTHD (r = 0.397, *P* < .01), highlighting the clinical relevance of psychological distress in PC, although the cross-sectional design and absence of longitudinal follow-up limit causal interpretation.^20^ Interventional studies suggested short-term improvements in mental health outcomes following MIL-centered approaches. Fraguell-Hernando et al. reported statistically significant reductions in depression (*P* = .001, d = 1.27) and anxiety (*P* = 0.017, d = 0.73) among participants receiving IMCP-PC, whereas no comparable changes were observed in the control group.^22^ Similarly, Kissane et al. reported moderate-to-large effect sizes in reducing depression (*P* = .008, d = 0.6) and demoralization (*P* = .003, d = 0.7) following “Meaning and Purpose” therapy.^24^ However, both trials were rated as high risk of bias due to small sample sizes, lack of blinding, and substantial attrition, which may have inflated effect estimates.^22,24^

Overall, the certainty of evidence for mental health outcomes was rated as very low using GRADE, reflecting serious concerns related to risk of bias, imprecision, and potential publication bias (Table 6). While the findings are consistent with a possible protective role of MIL in relation to psychological distress, confidence in the magnitude and durability of these effects remains limited.

#### “Meaning in Life”-Centered Interventions and Wish to Hasten Death

Four studies examined associations between existential variables and the WTHD.^19,20,23,25^ Bernard et al. found a significant inverse correlation between SWB and WTHD (*P* < .001),^25^ indicating that higher SWB was associated with lower endorsement of a WTHD. Guerrero-Torrelles et al. similarly found that depression and loss of meaning statistically mediated the relationship between physical impairment and WTHD.^20^ While this modeling highlights potential pathways linking physical and existential distress to WTHD, the observational design limits causal interpretation and may not account for unmeasured confounding.^20,25^ Testoni et al.^19^ further reported that higher MIL was associated with a reduced tendency to conceptualize death as annihilation (r = −0.62, *P* < .05), a cognitive representation that has been linked to attitudes toward death and dying. However, these findings were derived from a small sample (n = 30), substantially limiting statistical power and precision.^19^ Liu et al. also observed inverse correlations between dignity-related distress and MIL (r = −0.568, *P* < .01), suggesting an association between perceived meaning and existential suffering.^23^ Interpretation of these findings should consider the cultural and healthcare context of the study population, which may limit generalizability.^23^

Across studies, evidence regarding WTHD is derived exclusively from correlational or model-based analyses, precluding conclusions about directionality or intervention effects. Applying the GRADE framework, the certainty of evidence for WTHD outcomes was rated as very low, reflecting serious concerns related to indirectness, risk of bias, imprecision, and potential residual confounding (Table 6). Collectively, the findings are consistent with a possible buffering role of MIL and SWB in relation to WTHD; however, stronger longitudinal and interventional evidence is required to substantiate this hypothesis.

#### “Meaning in Life”-Centered Interventions and Existential/Spiritual Well-Being

Six studies explored associations between MIL and existential or SWB.^19–22^^,24,25^ In cross-sectional analyses, Bernard et al. found that SWB was significantly associated with both perceived meaning and overall QOL (*P* < .001),^25^ while Testoni et al. found that religious affiliation correlated with higher MIL scores (*P* < .001), suggesting that spiritual frameworks may contribute to existential grounding.^19^ Guerrero-Torrelles et al. identified loss of meaning as a mediator between physical impairment and existential distress,^20^ further underscoring the potential role of meaning in shaping psychological responses to illness. Gravier et al., in a cross-cultural sample, reported that spiritual pain was a key correlate of MIL, though the study did not explore cultural variations in detail.^21^

Intervention studies offered additional, though limited, insight. Fraguell-Hernando et al. reported that participants receiving IMCP-PC experienced a significant increase in perceived meaning (*P* < .01),^22^ and Kissane et al. observed improvements in SWB and life attitude following “meaning and purpose” therapy (*P* < .001 and *P* = .007, respectively).^24^ However, both RCT were rated at high risk of bias due to high attrition and lack of blinding,^22,24^ and small sample sizes further reduced the certainty of effect estimates. Cross-sectional studies,^19,20,25^ and Gravier et al.'s observational design^21^ also limit the ability to infer causality or directionality.

Applying the GRADE approach, the certainty of evidence for existential and SWB outcomes was rated as very low, due to serious concerns about risk of bias, methodological heterogeneity, and limitations in study design (Table 6). While conclusions must remain cautious, the overall pattern of associations across diverse contexts suggests that existential and spiritual dimensions are consistently linked with perceived meaning and may be relevant targets for MIL-centered care.

### Certainty of Evidence

Certainty of evidence for quantitative findings ranged from low (QOL, WTHD, SWB) to very low (mental health outcomes), primarily due to high risk of bias, imprecision, and potential publication bias.

The sole qualitative study, Rullán et al.,^18^ was appraised using GRADE-CERQual, resulting in moderate confidence in its core findings about the therapeutic and existential value of “Patient Dignity Inventory”-facilitated conversations.

## Discussion

### Summary of Evidence

This systematic review synthesized empirical evidence on the role of MIL in PC settings. Eight studies met inclusion criteria, spanning diverse methodologies: four cross-sectional studies, two RCT, one longitudinal observational study, and one qualitative study. The total sample included 1733 participants, the majority of whom were patients with advanced cancer.

Several studies reported associations between MIL-centered interventions and outcomes such as improved QOL, reduced emotional distress, enhanced existential and SWB, and decreased WTHD. However, these findings must be interpreted with caution. The empirical literature remains limited and heterogeneous, with marked variation in how MIL is conceptualized, operationalized, and integrated into interventions. Moreover, only two studies employed randomized designs, both of which were rated at high risk of bias. Observational and cross-sectional studies comprised the majority of the evidence base, restricting causal inference.

While the findings tentatively suggest that attending to MIL may hold value in PC, the overall certainty of the evidence was judged to be very low, particularly across quantitative outcomes. Our application of GRADE and GRADE-CERQual highlighted methodological shortcomings, including small sample sizes, lack of blinding, high attrition, and unmeasured confounding. These limitations constrain the strength of any conclusions regarding effectiveness. Although studies were conducted across diverse cultural settings, differences in the conceptualization and measurement of MIL were insufficiently examined, precluding meaningful cross-cultural comparison.

Nevertheless, the review highlights a consistent thematic signal across diverse settings: MIL and existential concerns are relevant to the lived experience of patients with advanced cancer. Future research should prioritize conceptually coherent interventions, methodologically robust designs, and longitudinal evaluations to clarify causal pathways and inform holistic, person-centered models of care at the EOL.

### General Interpretation of Results

The evidence synthesized in this review *suggests that MIL may be an important contributor* to QOL in PC, but the overall strength of this evidence remains limited. Interventions focused on MIL yielded improvements in overall well-being and QOL.^29^ These findings are partially supported by the wider literature. A meta-analysis by Dietrich et al.^30^ confirmed that meaning-centered psychotherapies have a large effect on improving QOL. This connection is further reinforced by evidence that MIL exerts a significant indirect effect on QOL through pathways involving SWB and depression in patients with terminal cancer, underscoring its potential role in enhancing EOL experiences.^31^ Similarly, family and interpersonal relationships were identified as key sources of MIL among Spanish patients with advanced cancer, contributing to higher satisfaction and QOL.^32^ In addition, a positive correlation between spiritual transcendence, MIL, and global QOL was observed in Polish EOL cancer patients, highlighting the universality of meaning as a fundamental human need across diverse cultural contexts.^33^ This connection is so fundamental that foundational frameworks like Ferrell's QOL model explicitly include the spiritual domain—encompassing meaning—as a pillar equal in importance to physical and psychological well-being.^29^

This review highlights *MIL's possible role as a buffer against psychological distress*, particularly depression,^19^ anxiety,^19^ and demoralization.^24^ The significant impact on demoralization is especially noteworthy, as it aligns with the work of Dong et al. and others who identify demoralization—a state of existential anguish and hopelessness—as a primary target for MIL-based therapies.^34^ A broader meta-analysis by Izgu et al. reinforces this, concluding that spiritual interventions, often centered on meaning, are effective in reducing psychological distress in this population.^35^ “Meaning of Life” Therapy, a culturally adapted intervention, elicited themes of family, identity, and reconciliation, effectively addressing psycho-existential needs and reducing distress in Portuguese PC patients.^36^ Among Chinese lung cancer patients undergoing radiochemotherapy, MIL was significantly associated with lower psychological distress, with mood state and social support identified as key contributing factors.^37^ Demoralization, marked by hopelessness and a loss of meaning, was reported in 13%–18% of cancer patients and was significantly linked to inadequately treated depression and anxiety.^4^ Meaning-Centered Group Psychotherapy significantly reduced depression and anxiety scores in patients with advanced urological tumors, further reinforcing the therapeutic potential of MIL-focused interventions.^38^ While these findings are promising, our GRADE analysis rated the *certainty of evidence for mental health outcomes as very low*, requiring cautious interpretation.^4,5^

A critical finding of this review is that a *stronger sense of meaning may help mitigate the WTHD* by addressing its existential roots. The study by Guerrero-Torrelles et al. illustrates this mechanism, showing that the loss of MIL was the most significant mediator between physical impairment and the WTHD.^20^ This suggests that it is not physical decline itself, but the resulting loss of purpose, that may drive the WTHD. This is corroborated by Bernard et al., who found a significant inverse correlation between SWB and WTHD,^25^ and Liu et al., who linked dignity-related distress—a proxy for lost meaning—to higher WTHD.^23^ These findings echo the conclusions of major reviews by Rodríguez-Prat et al. and Monforte-Royo et al., which identify existential distress as a more potent driver of WTHD than physical symptoms.^39,40^ Spanish patients with advanced cancer prioritized interpersonal relationships as key sources of MIL, which may foster a sense of connection and purpose.^32^ Consequently, MIL-centered interventions, by aiming to restore a sense of purpose, dignity, and value, *may play a role in reducing* WTHD. A recent systematic review on the WTHD in patients with life-limiting conditions identified depression, pain, functional impairment, diminished sense of MIL, perceived burden on others, and reduced QOL as the most commonly reported associated factors.^41^ Nonetheless, our review rated the overall *certainty of evidence for WTHD as very low*, limiting the strength of conclusions.

Finally, this review affirms that *MIL and SWB are deeply intertwined and mutually reinforcing constructs*. The multinational study by Gravier et al. found that spiritual pain was a significant negative predictor of MIL.^21^ Conversely, interventions focused on meaning were shown to improve SWB.^24^ Rullán et al. observed that the very process of discussing dignity and meaning was inherently therapeutic and enhanced SWB.^18^ A meta-analysis by Bauereiß et al.^42^ confirmed that existential interventions have a moderate positive effect on SWB (g = 0.52). “Meaning of Life” Therapy, which incorporates elements such as forgiveness and legacy documents, significantly enhanced SWB by addressing existential needs in PC patients.^36^ SWB and MIL were shown to significantly mediate the impact of depression on QOL, reinforcing their interconnected roles.^31^ Spirituality and religion were identified as important sources of MIL, with the overall sample reporting high satisfaction scores, suggesting both cultural and developmental influences on SWB.^43^ Additionally, 86% of advanced cancer patients endorsed spiritual concerns; thinking about meaning was significantly associated with poorer psychological QOL, yet addressing these concerns was considered critical for improving both SWB and overall QOL.^44^ These findings support the framework proposed by Ferrell, which underscores that addressing spiritual needs and sources of meaning is not optional but a fundamental, evidence-based component of PC.^45^ Although spiritual care is widely recognized as a cornerstone of PC, many healthcare professionals report difficulties in integrating it into clinical practice. The PALliatiVE approach offers practical guidance by summarizing key strategies into five core clinician attitudes: Prepare (P), Ask (A), Listen (L), Validate (V), and consult an Expert (E). This model may serve as a useful tool to support the delivery of meaningful and compassionate spiritual care.^46^ That said, our GRADE and GRADE-CERQual *ratings for outcomes related to SWB and existential well-being were also very low*, suggesting that current evidence—while thematically consistent—must be interpreted with caution.

### Limitations

The principal limitation of this review is that its conclusions are necessarily based on a small and methodologically weak body of evidence. Only eight studies met inclusion criteria, and the majority were affected by substantial design limitations. Only two studies were RCT, both of which were rated as having a high risk of bias, primarily due to non-blinded outcomes and high attrition. Most included studies were cross-sectional, precluding conclusions about causality or long-term effects. Sample sizes varied considerably, with several studies involving fewer than 50 participants, limiting statistical power and increasing the risk of imprecise effect estimates. These limitations were reflected in our GRADE assessments, which rated the certainty of evidence for all quantitative outcomes as very low.

In addition, there was marked heterogeneity in how MIL was conceptualized and measured across studies. A wide range of instruments was used, each capturing different dimensions of meaning. This variability complicates comparisons across studies and substantially limits the feasibility of quantitative synthesis (eg, meta-analysis). Furthermore, outcomes were predominantly self-reported, which may be influenced by social desirability bias or by patients’ psychological state at the time of assessment. Nevertheless, self-report measures remain standard in existential research, given the inherently subjective nature of constructs such as MIL and SWB.

Several review-level limitations should also be acknowledged. Although the review was conducted rigorously and followed PRISMA 2020 guidelines, the search was restricted to four databases and to English-language publications from 2016 to 2024. This may have excluded relevant studies published earlier or in other languages, introducing potential language and publication bias. The search strategy was developed by the authors without the involvement of an information specialist or medical librarian, which may have affected comprehensiveness. Grey literature, conference abstracts, and dissertations were not included; while this may enhance internal validity, it further reduces the breadth of the evidence base.

Finally, due to substantial heterogeneity in study design, populations, and outcome measures, no meta-analysis could be conducted. Although narrative synthesis was appropriate, it is inherently more subjective and less robust than quantitative approaches. The application of GRADE and GRADE-CERQual frameworks was performed post hoc and was not pre-specified in the PROSPERO registration; while these assessments improve interpretability, their retrospective application represents an additional limitation.

### Implications for Practice, Policy, and Future Research

The findings of this review suggest that existential suffering, including concerns related to MIL, is a relevant dimension of patient experience in PC. However, given the very low certainty of evidence identified using GRADE and GRADE-CERQual, these findings should be interpreted cautiously. At present, the evidence supports awareness and recognition of existential distress rather than the routine implementation of MIL-centered interventions. Healthcare professionals may benefit from training to identify existential concerns and to initiate supportive conversations or appropriate referrals, particularly when patients express distress related to meaning, purpose, or legacy. Brief, structured conversations about meaning may be acceptable and potentially supportive, but their therapeutic impact remains insufficiently established.

Therapeutic models such as IMCP-PC and “meaning and purpose” therapy have shown promising associations with improvements in existential and psychological outcomes in small and methodologically limited studies. However, the current evidence base does not justify their integration into routine care, and their use should remain confined to research settings or specialized services until more robust evidence becomes available.

From a policy perspective, this review does not support immediate changes to clinical pathways or mandates for implementation. Rather, it underscores the importance of recognizing existential and spiritual dimensions as areas warranting further investigation within PC. Policies should prioritize support for high-quality research, training initiatives aimed at improving clinicians’ competence in addressing existential distress, and the development of culturally sensitive frameworks to guide future intervention design.

This review emphasizes the need for more rigorous trials on MIL-centered interventions in advanced illness. Future studies should: Use larger, diverse samples and longitudinal designs to examine the sustainability of benefits; Standardize outcome measures to allow for comparability and meta-analysis; Explore mechanisms of change in meaning-centered therapy; Examine cultural factors influencing the experience and relevance of MIL; Evaluate the feasibility, cost-effectiveness and scalability of interventions in real-world PC settings.

Addressing these gaps is essential before meaningful clinical or policy recommendations can be made.

## Conclusions

This systematic review identified consistent associations between MIL and key psychological and existential outcomes in patients with advanced cancer receiving PC. Across the eight included studies—most of which were small, affected by high attrition, and methodologically limited—MIL was reported to be inversely associated with depression, anxiety, demoralization, and WTHD, and positively associated with QOL and SWB. These patterns emerged across diverse clinical and cultural contexts, suggesting that MIL may be a relevant and potentially beneficial focus of care in serious illness.

However, the overall certainty of evidence was very low. Findings should therefore be interpreted as preliminary and hypothesis-generating. At present, MIL support remains a promising but unproven adjunct to comprehensive PC, rather than an established standard of care. The results underscore the urgent need for high-quality research, including adequately powered trials and longitudinal studies, to determine the effectiveness, mechanisms, and appropriate implementation of MIL-centered interventions.

Addressing patients’ need for meaning, dignity, and purpose may hold therapeutic potential, but more rigorous evidence is needed before existential care can be fully integrated into routine PC practice.

## Key Message

In this systematic review (n = 8), fostering MIL in palliative care correlated with higher quality of life, lower emotional distress, greater existential and spiritual well-being, and a reduced wish to hasten death, highlighting the urgent need for standardized tools to assess meaning.

## Supplemental Material

sj-docx-1-pal-10.1177_08258597261426760 - Supplemental material for Meaning in Life in Palliative Cancer Care: Psychosocial and Existential Outcomes—A Systematic Review Supplemental material, sj-docx-1-pal-10.1177_08258597261426760 for Meaning in Life in Palliative Cancer Care: Psychosocial and Existential Outcomes—A Systematic Review by Francisco Rodrigues-Fouto and Paulo Reis-Pina in Journal of Palliative Care

## List of Abbreviations and Acronyms

EOL: End-of-Life;
IMCP-PC: Individual Meaning-Centered Psychotherapy-Palliative Care;
MIL: Meaning in Life;
PC: Palliative Care;
QOL: Quality of Life;
RCT: Randomized Controlled Trials;
SWB: Spiritual Well-Being;
WTHD: Wish to Hasten Death.
