# Supplementary material for: Meaning in Life in Palliative Cancer Care: Psychosocial and Existential Outcomes—A Systematic Review
Source: J Palliat Care. 2026 Feb 28;41(4):394–412. doi: 10.1177/08258597261426760 (PMC13550743; doi:10.1177/08258597261426760)
Supplement: Supplementary file 1 [file sj-docx-1-pal-10.1177_08258597261426760.docx]

## Supplementary Table 1 - Database search strategies

| **Database** | **Search string** | **Limits/Filters** |
| --- | --- | --- |
| MEDLINE (via PubMed) | (("palliative care"[All Fields] OR "terminal care"[All Fields] OR "hospice care"[All Fields] OR "end-of-life care"[All Fields]) AND ("cancer*"[All Fields] OR "malign*"[All Fields] OR "neoplasm*"[All Fields]) AND ("meaning in life"[All Fields] OR "purpose in life"[All Fields] OR "meaning of life"[All Fields] OR "purpose of life"[All Fields])) | MEDLINE;  Humans; Publication: 2016/01/01 to 2024/12/31; English; Adult: 19+ years |
| Scopus | ( TITLE-ABS-KEY ( ( "palliative care" OR "terminal care" OR "hospice care" OR "end-of-life care" ) ) AND TITLE-ABS-KEY ( ( cancer* OR malign* OR neoplasm* ) ) AND TITLE-ABS-KEY ( ( "meaning in life" OR "purpose in life" OR "meaning of life" OR "purpose of life" ) ) AND TITLE-ABS-KEY ( adult* ) ) | Humans; PUBYEAR: >2015 and <2025; English |
| Web of Science Core Collection | Results for ("palliative care" OR "terminal care" OR "hospice care" OR "end-of-life care") (Topic) AND (cancer* OR malign* OR neoplasm*) (Topic) AND ("meaning in life" OR "purpose in life" OR "meaning of life" OR "purpose of life") (Topic) AND adult* (Topic) | Timespan: 2016-01-01 to 2024-12-31; English |
| Cochrane Library | ("palliative care" or "terminal care" or "end-of-life care" or "hospice care") in Title Abstract Keyword AND (cancer* OR malign* OR neoplasm*) in Title Abstract Keyword AND ("meaning in life" OR "purpose in life" OR "meaning of life" OR "purpose of life") in Title Abstract Keyword AND adult* in Title Abstract Keyword (Word variations have been searched) | Publication: Jan 2016 to Dec 2024; English |
